# Plastic Population Effects and Conservative Leaf Traits in a Reciprocal Transplant Experiment Simulating Climate Warming in the Himalayas

**DOI:** 10.3389/fpls.2018.01069

**Published:** 2018-07-30

**Authors:** Haijun Cui, Joachim P. Töpper, Yan Yang, Vigdis Vandvik, Genxu Wang

**Affiliations:** ^1^National Plateau Wetlands Research Center, College of Wetlands, Southwest Forestry University, Kunming, China; ^2^Key Laboratory of Mountain Surface Processes and Ecological Regulation, Institute of Mountain Hazards and Environment, Chinese Academy of Sciences, Chengdu, China; ^3^University of Chinese Academy of Sciences, Beijing, China; ^4^Norwegian Institute for Nature Research, Bergen, Norway; ^5^Department of Biology, University of Bergen, Bergen, Norway; ^6^Bjerknes Centre for Climate Research, University of Bergen, Bergen, Norway

**Keywords:** climate change, alpine, reciprocal transplant experiment, altitudinal gradient, population growth rate, vital rate, *Viola biflora* var. *rockiana*, leaf traits

## Abstract

Climate warming poses considerable challenges for alpine plant species, especially for competitively inferior ones with resource-conservative adaptations to cold climates. The Himalayas are warming at rates considerably faster than the global average, so it is particularly important to assess how and through which mechanisms alpine plant species are affected there. We employed a demographic approach in a climate change experiment, where vegetation turfs were transplanted reciprocally between the central parts of the study species’ (*Viola biflora* L. var. *rockiana*) range and the warmer range margin, with a temperature difference of ca. 1°C. In addition, turfs were also transplanted outside the range to warmer habitats, simulating two different scenarios of climate warming, +1 and +4°C. Transplanting to warmer sites negatively impacted population growth rates (λ), survival and clonality, but did not affect growth and fecundity, while the productivity of the plant community increased. The reciprocal transplants to the colder habitat showed the opposite effects, for both *V. biflora* and the plant community, indicating plastic responses of the study species, driven by changes in plant–plant competition. However, the leaf traits underlying the modeled population growth rates were origin-site specific and not affected by the climate-change treatments over the study period, suggesting local adaptation of growth form to competition in the warmer range margin, and to climate adversity in the colder range center. The transplants outside the present species’ range showed consistently stronger reductions in population growth rate and survival, with mortality of 90–100% in the +4°C treatment. This illustrates that climatic changes beyond species’ present climatic ranges pose a serious risk for range contraction and extinction for Himalayan alpine species in the near future. As *V. biflora* seems mostly limited by competition under warming, its persistence in a future climate may become increasingly dependent on keeping competitive effects from the surrounding community low, for instance by management interventions like grazing and mowing.

## Introduction

Under global climate warming, especially alpine plant species with narrow ranges and locally adapted populations face a considerable risk of extinction (Holt, 1990; Theurillat and Guisan, 2001; Thomas et al., 2004). In contrast, in species where responses to climatic variability are plastic there may be time for evolutionary changes (Jump and Peñuelas, 2005). Thus, it is important to consider local adaptation and phenotypic plasticity of plant populations when estimating their vulnerability to climate warming (Matesanz et al., 2010; Nicotra et al., 2010). A powerful tool for studying local adaptation and phenotypic plasticity is the reciprocal transplant approach, where individuals of a focal species are transplanted reciprocally between two contrasting habitats within the species’ range. In such set-ups, opposed outcomes of the two transplant directions (i.e., negative effects in one transplant direction, positive effects in the other) indicate plasticity, whereas negative effects in both transplant directions indicate some degree of local adaptation (Claussen et al., 1940; Reznick and Travis, 1996; Kawecki and Ebert, 2004).

Environmental variation, and hence also experimental alteration, may affect various vital rates in a species’ life cycle, from germination (Levine et al., 2008) and survival probability (Simons et al., 2010), to flowering dynamics (Inouye et al., 2002; Pfeifer et al., 2006). Moreover, climatic effects on different vital rates often differ and may even be in opposed directions (Hutchings, 2010; Nicolé et al., 2011), so that even in cases where the overall population growth rate is not affected by climatic change, there may thus be strong effects on the underlying responses in vital rates. Detailed demographic analyses may therefore yield valuable information on environmental change impacts on plants beyond the directly observable effects on population size and abundance.

In this paper, we combine a reciprocal transplant experiment along an altitudinal gradient with a demographic study on a small alpine forb to investigate potential future responses of a typical alpine species under climate warming in the Gongga mountains in China. Vegetation turfs were transplanted from the species’ range center to its lower altitudinal range margin and vice versa. We collected demographic data on all individuals of *Viola biflora* L. var. *rockiana*, referred to as *V. biflora* from here on, in the experimental turfs to examine whether and how the species responds in both population abundance and underlying vital rates to a climatic change of ca. 1°C, the moderate regional 30-year prediction (Ding et al., 2007; Chen et al., 2013), within the species’ present altitudinal range. In order to explore impacts of warming on range edge populations and impacts of extreme warming, we complemented the reciprocal transplant setup with transplantations to beyond the species current climatic range, simulating warming of +1.4 and 3.9°C, respectively. This is especially relevant in the Himalaya region, which has experienced about twice the temperature rise as compared to the global average (Chen et al., 2013). *V. biflora* is a relatively weak competitor (Olsen et al., 2016), and we expect it to respond negatively to the warming treatments as the release from cold-temperature stress leads to higher productivity of competitive species in the turfs, more biomass and thus increased competition for light (cf. Hautier et al., 2009). We disentangle potential plastic responses from local adaptations by testing for opposite responses vs. all-negative responses of transplants in both directions (Kawecki and Ebert, 2004). Moreover, under plastic responses, we expect increasingly stronger detrimental effects in the +1.4 and +3.9°C transplants outside the species’ range than in the 1°C warming within the range.

## Materials and Methods

### Study Species

*Viola biflora* is a perennial, clonal forb species, which is common in snowbeds and leesides, grazed upland pastures, stream banks, and birch forests in the alpine and high alpine zone. It grows in open, relatively nutrient rich habitats with good access to moisture, and is characterized as a weak competitor (Evju et al., 2010).

### Study Sites

**FIGURE 1:**
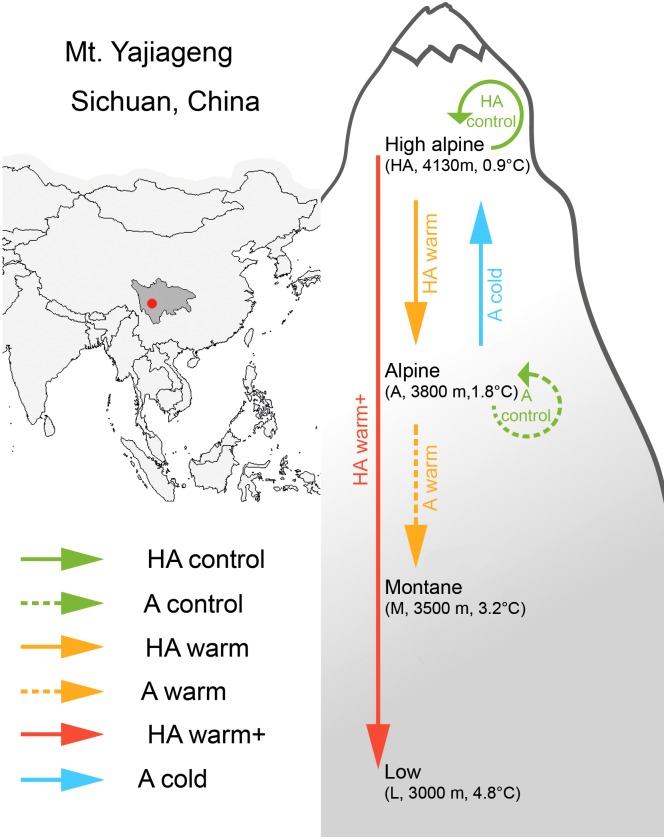
Geographic location and experimental setup for the transplant experiment. Arrows indicate direction of transplantation; for each of the four sites altitude and mean annual temperature are indicated.

The study was conducted at Mount Yajiageng on the eastern fringe of the Tibetan Plateau from 2012 to 2015. We selected four sites in the natural open grasslands along the western slope of the mountain at altitudes from 3000 to 4130 masl, a low site (L, 3000 m), a montane site (M, 3500 m), an alpine site (A, 3800 m), and a high alpine site (HA, 4130 m). Sites A and HA are located in the natural shrub and grass ecotone, where the study species, *V. biflora*, is common and relatively widespread. The vegetation at these sites is dominated by *Kobresia uncinioides*, *Kobresia royleana*, *Potentilla stenophylla*, *Saussurea ceterach*, *Saussurea stella*, and *Primula* spp. (site HA), and *Polygonum viviparum*, *Potentilla leuconota*, *Carex laeta*, and *Carex atrata* (site A). Sites L and M lie below the natural tree line, where the study species does not occur naturally. At these sites, the vegetation is dominated by *Deyeuxia scabrescens*, *Halenia elliptica*, *Pedicularis davidii*, *Pedicularis sima*, *Geranium pylzowianum*, and *Anaphalis nepalensis* (site M), and *Carex finitima*, *Carex nubigena*, *Plantago asiatica*, *P. leuconota*, *Trifolium repens*, and *Clinopodium polycephalum* (site L). The study area is located in the transition zone between subtropical humid monsoon climate of eastern China and cold climate of Tibetan Plateau, with a mean annual precipitation of ca. 1000–2000 mm/a concentrated in May to October (Gongga Mountain Alpine Ecosystem Observation Station; cf. Zhou et al., 2013). The four sites are characterized by a pronounced temperature gradient (**Figure 1**). While precipitation decreases along this altitude gradient as well, largely due to lower July precipitation in A and HA, air relative humidity and soil moisture do not vary systematically with altitude (Supplementary Table S1). All sites are characterized by mountain dark brown soil and the vegetation of the four sites is all upland grasslands under low-intensity extensive grazing from horses, cattle, sheep, and yak. The sites were fenced in July 2012 to avoid animal damage of the experimental plots. After fences were erected, the vegetation was cut annually to avoid biomass buildup.

### Experimental Design

In sites HA and A, we established five experimental blocks, and in each block three semi-randomly placed 25 cm × 25 cm plots (they had to contain the study species). In September 2012, two plots from each block of site HA were transplanted to site A and site L, representing a temperature change of +0.9°C within the species range and +3.9°C outside the species range, respectively. In addition, two plots from each block of site A were transplanted to site M and site HA, representing a temperature change of +1.4°C outside the species range and of -0.9°C within the range, respectively. For simplicity, we hereafter refer to these transplants as the “HA warm” (i.e., +0.9°C warming within the range), “HA warm+” (i.e., +3.9°C warming outside the range), “A warm” (i.e., +1.4°C warming outside the range), and “A cold” (i.e., -0.9°C cooling, from range margin to range center) treatment, respectively. The fifth plot was transplanted within the original site and block, as a control (we hereafter refer to the controls at HA and A as “HA control” and “A control,” respectively). The transplanted turfs measured 25 cm × 25 cm and were 20 cm deep. After excavation, the turfs were placed in wooden boxes and replanted into their respective target sites within 1 day.

### Data Collection

As a clonal plant, *V. biflora* produces long lateral rhizomes, each with multiple flowering ramets, on the same genetic individual. As the below-ground connections cannot be assessed under non-destructive sampling, we used ramets as our working unit (Hegland et al., 2010). In July/August 2012, prior to transplanting, we non-destructively marked all *V. biflora* ramets within each plot with toothpicks, measured a selected set of vegetative traits and counted the number of flowers and capsules. The vegetative traits “number of leaves” and “length of the longest leaf” were used to estimate plant biomass (hereafter referred to as “size”), based on a regression model tested on 236 destructively sampled individuals (outside the experimental plots) from sites HA and A in 2012 and 2013 (Supplementary Table S2). In the summers of 2013, 2014, and 2015, we recorded the survival of the previous year ramets, tagged new ramets and seedlings, and repeated the measures of vegetative and of reproductive traits for all life ramets. For *V. biflora* it is impossible to non-destructively assess how ramets are connected and new clones got therefore the closest ramet (but not seedlings) assigned as “parent.” *V. biflora* exhibits vegetative dormancy with ramets being able to resprout after having been dormant for up to 4 years (Evju et al., 2010). Therefore, the limited period of the study does not allow disentangling mortality from “going dormant” and clonality from “resprouting.” We hence regarded all newly appearing non-seedling ramets as clonal offspring and all disappearing ramets as “dead.”

To get an estimate of productivity and competition for light in the experimental sites and treatment plots we measured overall community height (see Hautier et al., 2009) and cover in 2012, prior to transplanting, as well as in 2013, 2014, and 2015. Community height was determined as the average of five measurements of the foliage height per plot, and cover was determined as the percentage of the plot covered by all vascular plant species.

### Statistical Analyses

#### Population Models for *V. biflora*

To assess the population dynamics of the study species and estimate population growth rates (λ) we used integral projection models (IPMs) (Easterling et al., 2000; Ellner and Rees, 2006, 2007). IPMs are continuous analogs of matrix models (Caswell, 2001) based on regressions of vital rates (survival, growth, clonality, and fecundity) against a continuous state variable (size, weight, age, etc.,) describing each individuals’ state (Easterling et al., 2000; Ellner and Rees, 2006, 2007; Merow et al., 2014; Rees et al., 2014). We used R version 2.15.3 (R Core Team, 2014) to build IPMs separately for each control and treatment population. For the vital rate regressions the IPMs are built from, we examined the effects of plant size in a given year on survival, size in the following year (i.e., growth), probability of producing clonal offspring, number of clonal offspring produced, size of clonal offspring, flowering probability, and number of flowers produced. This was done separately for each transplant treatment and the controls across all sites and transitions (i.e., the time between the annual censuses: 2012–2013, 2013–2014, and 2014–2015) using generalized linear mixed effects models (GLMMs, R-package lme4; Bates et al., 2014). In addition to assessing “size” as the deterministic “fixed effect,” these models allow specification of stochastic “random effects” enabling us to model the stochastic variation caused by the spatial structure of the experimental setup and the repeated measures on the same plants. All vital rate models were first fitted with linear terms for size in the fixed effects, as well as random intercepts and slopes for every combination of site and annual transition (SiteTrans in Supplementary Material S3). Additionally incorporating block and plot as random effects over-parameterized the models and lead to non-convergence, and we therefore dropped these random effects. The appropriate minimum model structure for both fixed and random effects was found with a backward selection procedure using likelihood ratio tests (significance level 0.05). In this procedure, we considered dropping the linear terms for size in the fixed effects, and the random slopes, whereas the random intercepts were always kept as the minimum random structure. The variables “number of clones” and “number of flowers” showed too little variability to warrant estimation of random effects and for these we thus used simpler generalized linear models instead. For the models on probability of survival, clonal reproduction, and flowering, we used a binomial error distribution with logit link, for the models on number of clonal offspring and flowers we used a Poisson error distribution with log link, and for the models on growth and size of clonal offspring we used a Gaussian error distribution with identity link. Where necessary, over-dispersion in the binomial and Poisson models was accounted for by extending the error structure with an observation-level random effect (Maindonald and Braun, 2006). More detailed documentation of the vital rate models can be found in Supplementary Material S3.

Some reproductive traits such as the number of seeds per flower, seedling establishment, the probabilities for entering and staying in the seed bank, and seedling size cannot be related to plant size under non-invasive data collection. These values were obtained from another study on the focal species in a climatically similar environment (Olsen et al., 2016) and used as constants in all models.

Using the R-package IPMpack (Metcalf et al., 2013) we built IPMs from the above-described regression models for the vital rates growth, survival, clonality (based on probability of producing clonal offspring, number of clonal offspring produced, and size of clonal offspring) and fecundity (based on flowering probability, number of flowers produced, number of seeds per flower, the probability of seed germination and seedling establishment and entering the seed bank, as well as the mean size of seedlings). The seed bank is a discrete stage in an otherwise continuous population model, and was represented by a model describing transitions between the continuous distribution of plant sizes and the discrete seed bank (probability of staying in the seed bank, leaving the seed bank with subsequent seedling establishment and leaving the seed bank with subsequent seedling establishment failure) (Metcalf et al., 2013). These five vital rate models were then used to construct growth-survival (P), clonality (C), and fecundity (F) matrices (the discrete transition seed bank model goes into the *P*-matrix) with size ranges from the observed minimum and maximum sizes minus/plus an increment of 1% of the minimum/maximum size as described in, e.g., Metcalf et al. (2013). The matrices were of the bin dimensions 101 × 101 with the first bin representing the seed bank transitions and the bins 2-101 representing the continuous part of the size range. Finally, these matrices were combined into a full IPM from which the dominant eigenvalue λ, representing population growth rate in population projection matrices, could be calculated. Separate IPMs were constructed for each transplant treatment and the controls in every site and every year.

We estimated the uncertainty around the λ’s by bootstrapping. Individual ramets were sampled with replacement to construct a resampled dataset containing the same number of observations as the original dataset. Regression modeling, construction of IPMs and calculation of λ were then repeated as described above using the resampled dataset. Performing this procedure 2000 times generated a set of bootstrap λ estimates, which were used to assess the significance of differences in λ between transplants and controls. Pairwise independent transplant and control bootstrap λ samples were subtracted from each other (control-treatment). A qualitative difference in λ was accepted as significant at the 0.05 level when it occurred in more than 95% of the bootstrap sample pairs.

Finally, we used life table response experiments (LTRE) to determine how much changes in the vital rates contributed to the differences in λ between the transplants and their respective controls. The contribution of a given vital rate was calculated as the sum of the differences between the vital rate matrices of the transplant and control treatments multiplied by the sensitivity of a matrix midway between the full IPM matrices of the two treatments (i.e., transplant and control) (Caswell, 2001). We separated growth and survival, which together make up the *P*-matrix, by setting the probability of survival to one for all sizes. The contribution of growth alone could then be calculated using the method outlined above. By subtracting the contribution of growth from the total growth-survival contribution we found the contribution from survival alone.

#### Leaf Traits of *V. biflora*

To address origin-related differences in plant architecture we assessed differences in number of leaves, leaf size, and length of the leaf stalk from all plants in all years in our study. For this, we modeled all three variables as a function of origin (fixed effect) and with random intercepts for all treatment–origin combinations and random intercepts for year. We used GLMMs with Poisson errors and log-link for number of leaves, and with Gaussian errors and identity-link for leaf size and leaf stalk length.

#### Community Responses

The difference in community height or cover between transplant plots and controls was analyzed for each transplant treatment by linear mixed effects models with Gaussian error structure. We used year as fixed effect to assess whether or not the difference observed in 2013, 2014, and 2015 were significantly different from prior to transplanting in 2012. The model intercept represents 2012 and indicates whether or not the observed difference in that year is significantly different from zero.

## Results

### Population Growth Rate (λ)

**FIGURE 2:**
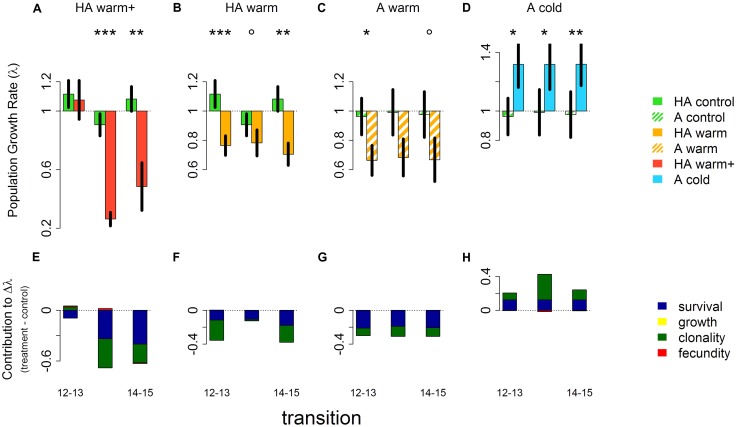
Population growth rates (λ) for each transition (i.e., annual transitions: 2012–2013, 2013–2014, and 2014–2015) in controls at the site of origin and transplants at the target sites **(A–D)** and contributions to differences in population growth rates between transplants and the respective controls at the site of origin **(E–H)**. Treatment colors in panels **(A–D)** match the colors in **Figure 1**. Error bars in panels **(A–D)** show ±1 standard deviation of λ from the bootstrapped datasets (*n* = 2200). Significant differences between λ in controls and transplants indicated by asterisks: ^∗∗∗^*p* < 0.001, ^∗∗^*p* < 0.01, ^∗^*p* < 0.05, °*p* < 0.1.

Both the high alpine and the alpine native populations of *V. biflora* were stable (λ ∼ 0.9–1.1 and λ ∼ 0.96–0.99, respectively) throughout the study period and all transplant treatments showed clear and significant differences from this stable state (**Figures 2A–D** and **Table 1**). Transplanting to warmer sites reduced λ in populations originating from both the high alpine and the alpine site, but the magnitude of the effect varied. λ was around 0.7–0.8 in the “HA warm” treatment, 0.7 in the “A warm” treatment, and as low as 0.3–0.5 in the “HA warm+” treatment. As the population size at site A was much smaller than at site HA (see **Table 1**) uncertainty was higher in the transplants originating from site A, potentially masking statistical significance of the effects of the “A warm” treatment. The transplants from the alpine site to the colder high alpine site led to a λ increase of ∼30% which was significant for all transitions. In the “HA warm+” transplants to the low alpine site, λ was unchanged in the first transition but then dramatically dropped in the following years.

**Table 1:** Population sizes in all transplant treatments and sites in the four study years.

Year	HA control	A control	HA warm	A warm	HA warm+	A cold
2012	98	16	121	21	75	23
2013	127	13	92	14	87	21
2014	106	12	69	9	18	38
2015	131	11	59	8	10	54


*HA and A depict the origin of the transplants, i.e., high alpine site and the alpine site, respectively.*

### Vital Rates

Changes in survival and clonality were the main contributors to differences in λ in all treatments (**Figures 2E–H**). Similarly to λ, the negative survival contributions in the warming treatments increased progressively from the “HA warm” treatment, via the “A warm” treatment and to the “HA warm+” treatment. The “A cold” and the “HA warm” treatments were complementary with respect to survival, the respective positive and negative contributions to changes in λ being comparable in effect size.

Growth did not significantly contribute to changes in lambda between treatments, while fecundity slightly increased in the “HA warm+” transplants and slightly decreased in the “A cold” transplants.

### Height and Cover of the Plant Community

**FIGURE 3:**
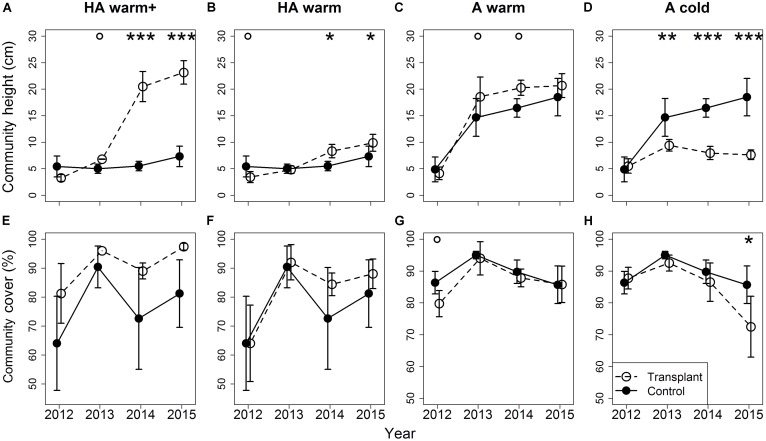
Differences in community height **(A–D)** and cover **(E–H)** between transplants at the target sites and controls at the site of origin. Error bars indicate 95% confidence intervals. Significant differences between community height/cover in controls and transplants indicated by asterisks: ^∗∗∗^*p* < 0.001, ^∗∗^*p* < 0.01, ^∗^*p* < 0.05, °*p* < 0.1. Treatments are indicated by symbols: open symbols and dashed lines for transplants, filled symbols and solid lines for controls at the site of origin. The positions of the different time series are slightly shifted along the *x*-axis to facilitate readability.

Community height was higher under warming than in the controls in the course of the experiment, by ∼3 cm under both the “HA warm” and the “A warm” treatments (**Figures 3B,C**), and by ∼16 cm in the “HA warm+” transplants from the second transition on (**Figure 3A**). In the “A cold” transplants, community height was lower than in the controls by ∼5 cm already in the first transition, the effect size increasing in the following transitions to ∼10 cm (**Figure 3D**). Cover was mainly unaffected by the climate treatments (**Figures 3E–H**), but was significantly lower in the “A cold” transplants in the last transition (**Figure 3H**). In site HA, cover was highly variable (ranging from 50 to 90%), whereas it was constant and high in the transplants (both to site A and site L) originating from this site (**Figures 3E,F**).

### Leaf Traits

**FIGURE 4:**
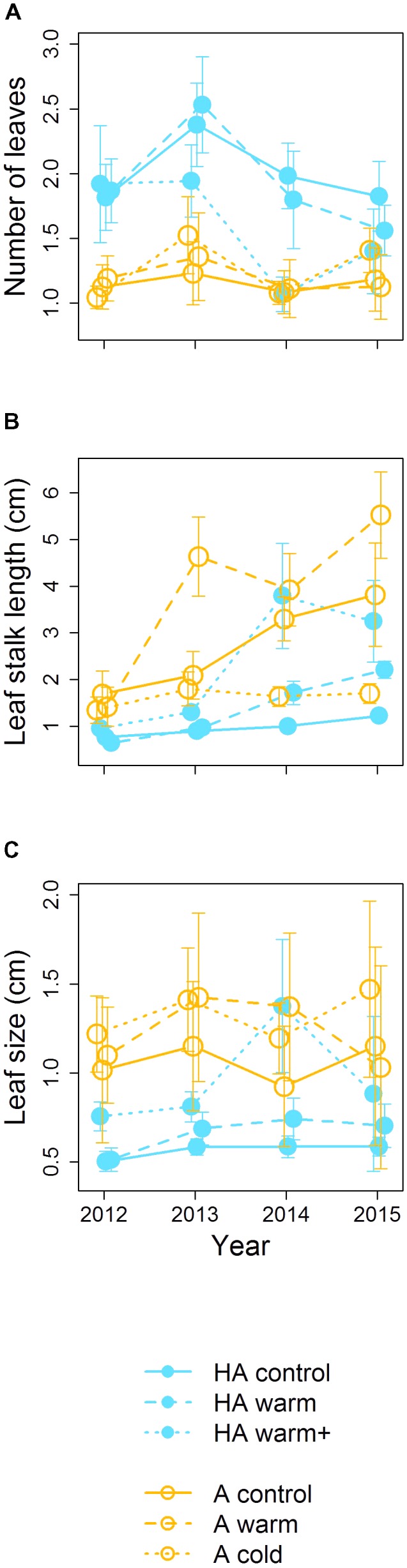
Leaf traits (**A–C**: Number of leaves, Leaf stalk length and Leaf size) in transplants (dashed and dotted lines) and controls (solid lines) from high alpine (blue, filled symbols) and alpine origin (orange, open symbols) over the study period. Error bars indicate 95% confidence intervals. The positions of the different time series are slightly shifted along the *x*-axis to facilitate readability.

Number of leaves, leaf size, and leaf stalk length had different values in the high alpine and alpine habitats. In the high alpine, the plants produced more, but smaller and lower growing leaves, and in the alpine the plants produced fewer, larger and higher growing leaves (**Figure 4** and **Table 2**). For number of leaves and leaf size, the transplanted populations retained their original trait values even after transplantation to climates differing with ∼1°C (**Figures 4A,C**). In the “HA warm+” treatment, however, these two traits changed from the “high alpine values” in direction of the “alpine values.”

**Table 2:** Differences in leaf traits between the high alpine (HA) and the alpine (A) sites.

Leaf trait	Effect (HA–A)	SE	*N*	*p*-Value	*SD* treat	*SD* trans
# Leaves	0.40	0.10	876	<0.001	0.08	0.13
Leaf-stalk length	-1.53	0.96	876	0.19	1.17	0.40
Leaf size	-0.54	0.13	868	0.01	0.13	<0.001


*Shown are the regression statistics on the differences (effect) in number of leaves (# leaves), leaf-stalk length, and leaf size between the high alpine (HA) and the alpine (A) sites, as well as the associated standard errors (SE), sample sizes (*N*), and *p*-values. In this analysis, the leaf trait data for all treatments and years were lumped together and analyzed with specifying random intercepts for the different treatments (HA control, A control, HA warm, A warm, HA warm+, and A cold). SD treat and SD trans indicate the standard deviation among treatments and transitions, respectively.*

## Discussion

The results of this transplant study clearly show negative effects of warming on the population dynamics of the small herb *V. biflora*. This contrasts the general positive impacts on plant growth and productivity commonly found as a response to warming (Myneni et al., 1997; Rustad et al., 2001; Walker et al., 2006; Wu et al., 2011; Elmendorf et al., 2012). These positive impacts are associated with direct physiological effects of higher temperatures on photosynthesis (Sage and Kubien, 2007), although warming-caused drying may reverse these impacts (Barber et al., 2000; Ciais et al., 2005). However, a general increase in productivity often leads to a shift in dominance structure in the vegetation (Harte and Shaw, 1995), penalizing species with a resource-conservative, cold climate strategy that cannot utilize the higher temperatures effectively (Klanderud and Totland, 2005; Klanderud et al., 2015). *V. biflora* is a relatively weak competitor for light (Evju et al., 2010), and therefore we suggest that the observed impacts are indirect effects via increased competition from the more productive plant community under warming (**Figure 3**), although we acknowledge that our study design does not explicitly test for indirect competition effects vs. direct climate effects. Higher vegetation canopies reduce light availability in the vegetation sward and thus penalize smaller, less competitive species (Hautier et al., 2009). In line with this, we found changes in growth to play an inferior role in *V. biflora*, while changes in survival and clonal growth constituted the main drivers of changes in population growth rates. Decreased survival and clonality, without any delay via prior changes in plant sizes, translate into swift reductions on population size, which in turn increases the chance for local extinctions of *V. biflora* populations (cf. Morris and Doak, 2002), especially in the species’ warmer range margin (cf. Hampe and Petit, 2005).

### Plasticity vs. Local Adaptation

The results from our reciprocal transplant treatments, ca. +1°C warmer and -1°C colder for high alpine and alpine populations, respectively, clearly show that both populations perform better in the high alpine habitat than in the alpine. The opposite effects of warming and cooling on lambda with similar effect sizes and similar suites of vital rate contributions to changes in lambda support neither the “local vs. foreign” or “home vs. away” criterion for detection of local adaptation and hence suggest a plastic response to altered temperature (Kawecki and Ebert, 2004). Nevertheless, the control populations were similarly stable (λ ∼ 1) in both habitats. This seemingly contradicts the plastic effects found in the reciprocal transplants, from which we could have expected lower fitness in the alpine population than in the high alpine population. However, this discrepancy can make sense in the light of the stress-gradient-hypothesis (Choler et al., 2001; Pugnaire and Luque, 2001; He et al., 2013; Klanderud et al., 2017) which suggests that plant species are rather limited by cold temperatures at the adverse end of their temperature range but by competition at the favorable end of their temperature range. At both ends any established populations would persist at locally suitable habitats that are not too harsh in the high alpine and not too competitive in the alpine. When transplanted, however, a release from competition seems to be positive in spite of a harsher climate in the high alpine, whereas an increase in competition seems to be negative in spite of a more favorable climate in the alpine.

These two limitations are reflected by different growth forms of *V. biflora* individuals in the alpine and high alpine habitats in our study (**Figure 4**). In the high alpine sites, the plants produce several, small and low standing leaves, a classical growth form strategy in climatically harsh environments (Larcher, 2003); in the alpine site, the plants grow fewer, but larger leaves on higher leaf stalks, a good strategy under higher competition for light (Weiner and Thomas, 1992; Weiner, 2004; Poorter et al., 2012). Both leaf size and number of leaves were very conservative in our data, with individuals in both the controls and the transplants originating from the same site sharing similar values despite the temperature difference after transplantation. This indicates that these leaf traits in *V. biflora* do not change plastically with climate but are at least to some degree adapted to the relevant local stressors, climatic adversity and competition. We have no information about the actual biomass associated with size of leaves and leaf stalks and we thus do not know whether the observed differences reflect patterns in allocation or organ morphology, but plants have been shown to be less able to adjust allocation than organ morphology (Poorter et al., 2012). Based on these results, one could expect that transplanted populations failing to acclimate their individuals’ growth form to the locally prevalent stressors should be penalized. However, in our study this was only the case for the small growing individuals from the high alpine transplanted into the alpine habitat, whereas the taller growing individuals from the alpine habitat thrived better when transplanted to the high alpine, even when compared to the local high alpine controls. This could suggest that climatic adversity is a less limiting factor in our study sites than competition, but without any further experimental evidence to support such a conclusion, this remains speculative.

In a climate change perspective, conservative leaf traits may be rather detrimental, as this prevents plastic adjustments to a higher vegetation under a warmer climate and hence causes range contractions (Anderson et al., 2009; Jones and Gilbert, 2016). However, under the extreme “HA warm+” treatment, number of leaves and leaf size seemingly displayed less conservatism than indicated by the other treatments, as their values changed in the 3 year (**Figures 4A,C**). From the second transition (i.e., 2nd to 3rd year) on, mortality in this treatment was very large, reaching 94 and 100% in the last two transitions. Hence, the remaining population in the last 2 years consisted mostly of new recruits, which adopted the above indicated altered growth form as an ontogenetic acclimatization to their environment (Mason et al., 2013). Although mitigation of negative effects at the species level is still deemed unlikely for alpine plants especially under stronger warming scenarios (Parmesan, 2006), this may facilitate local persistence and acclimatization to the environmental conditions in the short-term or under moderate climate change. In species with clear patterns of local adaptation, as, e.g., *Erysimum capitatum* in North America (Kim and Donohue, 2013), regional persistence under climate warming would need to rely on niche-tracking and gene-flow, or ultimately evolutionary adaptation.

### Warming Within the Temperature Range vs. Outside the Range

Warming that takes the plants outside their present temperature range caused a stronger decrease in lambda based on stronger reductions in survival when compared to the same temperature rise within the range. This mirrors the results from a study in Norway, where populations of *V. biflora* from the lower altitudinal range margin performed worse than populations from the range center when transplanted to sites with warmer and wetter climate (Töpper et al., 2018). The concurrent increase in community height, however, was similar under ca. 1°C warming both within and outside the species range in our study. This suggests that populations growing in the leading range margin of an environmental stressor (here, competition in alpine habitats) experience an increase in that stressor more adversely than more central-range populations (Anderson et al., 2009; Hargreaves et al., 2014).

In line with Gavazov et al. (2014), who found that warming of +4°C had more negative impacts on sub-alpine pastures in Switzerland than more moderate warming, the extreme “HA warm+” transplants in our study showed a considerably lower lambda in *V. biflora* than in the controls based on even more reduced survival. However, these reductions showed a lag of 1 year, which was not present in the other, more moderate, warming treatments. Interestingly, change in plant–plant competition, expressed as change in community height, also showed a lagged increase, probably due to a general (i.e., for all species) need to adjust the photosynthetic temperature optimum under warming of 4°C (Yamori et al., 2014). Thus, the *V. biflora* population did not experience an increase in competitive stress during the first transition, which may explain how the population could remain relatively stable in the 1st year after transplantation. Nevertheless, once the community height increased, this increase was drastic and likewise was the reduction in survival and lambda. With only 10 individuals left in 2015 (all clonal recruits), the persistence of this *V. biflora* population at the low site is highly unlikely (Morris and Doak, 2002). While no alpine species has yet got extinct from the transplanted turfs in our study, colonization from the surrounding local vegetation has occurred frequently (Yang et al., unpublished). These “novel competitors” may constitute a significant part of the community height increase and thus contribute to shaping the responses of alpine species like *V. biflora* (Alexander et al., 2015).

During this lagged response phase, changes in clonality were, surprisingly, contributing positively to the difference in lambda. In our study, such increase in clonality may also be due to increased re-sprouting after vegetative dormancy. Such re-appearance of dormant plants has been linked to re-mobilization of stored resources (Gremer et al., 2010), which may be interpreted as a stress response contributing to the apparent stabilization of this *V. biflora* population during the first transition. As both dormancy and clonality may be susceptible to effects of climate change (Carlsson and Callaghan, 1994; Symstad and Tilman, 2001), a separation of dormancy and clonal reproduction based on a longer term study would be enlightening.

### Limitations

While yielding many important insights into climate-change driven responses on alpine plants, our study also has two limitations worth discussing. First, as mentioned above, vegetative dormancy is a factor complicating non-destructive population studies. If the demographic time series is long enough and the temporal extent of the dormant phase short enough, then dormancy can be identified and modeled with satisfactory levels of uncertainty (Kery et al., 2005; Evju et al., 2010). In short-term studies, as the one at hand, identification of dormant stages is impossible for most cases of “missing” observations: is a new individual in year 2 a clone or a previously dormant plant? Is an individual missing the last year dead or dormant? These questions consider 1-year dormancy, which hence would complicate demographic studies over up to four transitions, but 2- and 3-year dormancy is just as common (Spindelböck and Olsen, 2013), ultimately increasing the required number of transitions for reasonably sound inference on dormancy. The approach adopted in this study, ignoring dormancy by treating all missing individuals as dead and all re-appearing individuals as clones (Olsen et al., 2016; Töpper et al., 2018), constitutes a simplification of the species’ life cycle and ecology. However, while this needs to be considered when dealing with vital rate contributions, lambda still reflects the population-size dynamics above ground, which still harbors the crucial life stages for assessing ecological performance as green plants failing to re-sprout also increase their risk to die (Shefferson, 2006).

Second, our experiment is replicated locally but not regionally, which in principle limits the degree to which our results can be generalized beyond the mountain our study was performed on to alpine systems in general. However, our overall finding of competition-limitation in *V. biflora* is well supported by other population studies on this species from Europe (Evju et al., 2010; Olsen et al., 2016; Töpper et al., 2018), and the responses found in our study thus do not seem to be “locally special” for Mountain Gongga.

## Conclusion

The average regional predictions for climate warming in China lie around 1-2°C until about 2050, while extreme models predict an increase by more than 4°C in the second half of the 21st century (Ding et al., 2007), which is double the climatic change predicted for the planet on average (Chen et al., 2013). This study illustrates how these scenarios may affect alpine herbs in the region and elaborates on the underlying mechanisms of population change. Our results show that the study species, the alpine herb *V. biflora*, clearly is sensitive to climate warming, which is threatening especially populations at the warmer species range margin, where competition already acts limiting for survival and establishment of new recruits in the species. There, the species’ persistence will likely become increasingly dependent on reducing competition from the plant community, e.g., through management interventions like grazing and mowing (Evju et al., 2010). Should the global warming trajectory follow the worse scenarios, also more central populations are at high extinction risk during this century, as these climatic changes would take the populations well out of their present environmental range.

## Author Contributions

VV, YY, and GW conceived and designed the experiment. JT designed the demographic study. HC and YY performed the experiment with input from VV. GW and YY provided climate data. JT and HC analyzed the data. JT wrote the paper with contributions from HC and VV. All the authors read and approved the final manuscript.

## Conflict of Interest Statement

The authors declare that the research was conducted in the absence of any commercial or financial relationships that could be construed as a potential conflict of interest.
