# Supplementary material for: Plastic Population Effects and Conservative Leaf Traits in a Reciprocal Transplant Experiment Simulating Climate Warming in the Himalayas
Source: Front Plant Sci. 2018 Jul 30;9:1069. doi: 10.3389/fpls.2018.01069 (PMC6077237; doi:10.3389/fpls.2018.01069)
Supplement: Supplementary file 1 [file Table_1.DOCX]

**Table S1** Annual and summer climate data in all four sites in 2013.

| Site | HA | A | M | L |
| --- | --- | --- | --- | --- |
| ANN-T, ℃ | 0.93 | 1.78 | 3.24 | 4.81 |
| SUM-T, ℃ | 7.65 | 8.87 | 10.63 | 12.73 |
| ANN-P, mm | 951.2 | 1124.8 | 1541.8 | 1708.2 |
| SUM-P, mm | 506 | 577.4 | 739.2 | 738.2 |
| ANN-SM, m³/m³ | 0.3 | 0.33 | 0.42 | 0.33 |
| SUM-SM, m³/m³ | 0.35 | 0.36 | 0.44 | 0.34 |
| ANN-RH, % | 76.63 | 81.88 | 84.94 | 87.03 |
| SUM-RH, % | 93.47 | 94.66 | 94.52 | 92.06 |

Note: ANN-T—annual mean air temperature; SUM-T—summer (July to August) mean air temperature; ANN-P—annual precipitation; SUM-P—summer (July to August) precipitation; ANN-SM—annual mean soil moisture at 5 cm depth; SUM-SM—summer mean soil moisture at 5 cm depth; ANN-RH—annual mean air [relative](javascript:void(0);) [humidity](javascript:void(0);); SUM-RH—summer mean air [relative](javascript:void(0);) [humidity](javascript:void(0);)
