# Supplementary material for: Plastic Population Effects and Conservative Leaf Traits in a Reciprocal Transplant Experiment Simulating Climate Warming in the Himalayas
Source: Front Plant Sci. 2018 Jul 30;9:1069. doi: 10.3389/fpls.2018.01069 (PMC6077237; doi:10.3389/fpls.2018.01069)
Supplement: Supplementary file 2 [file Table_2.DOCX]

**Table S2** Biomass regression table based on 236 individuals from sites A and HA in 2012 and 2013, that were harvested, dried and weighed after taking the demographic measurements. Shown are (a) the fixed effects from a linear mixed-effects regression model (lmer) taking into account the spatial (sites A and H) and temporal (censuses from 2 years) variation in biomass, (b) the associated random effects for Site, Year and residuals, and (c) the ANOVA-table from the linear mixed-effects model. The marginal R^2^ (associated with fixed effects only) and the conditional R^2^ (associated with full model) for this model are 0.55 and 0.77, respectively.

| **(a) Fixed effects** | coefficient | SE | p |
| --- | --- | --- | --- |
| β_0_ (intercept) | 0.71 | 0.43 | 0.19 |
| β_1_ (# leaves) | 0.29 | 0.02 | < 0.001 |
| β_2_ (length of longest leaf in mm) | 1.71 | 0.12 | < 0.001 |

Note: The biomass of an individual *i* then calculates as:

$$\mathrm{biomass}_{i}\sim\beta_{0}+{\#leaves}_{i}*\beta_{1}+{length\_longest\_leaf}_{i}*\beta_{2}$$

| **(b) Random effects** | variance | SD | N |
| --- | --- | --- | --- |
| Site | 0.13 | 0.37 | 2 |
| Year | 0.21 | 0.46 | 2 |
| Residual | 0.36 | 0.60 | 236 |

| **(c) lmer ANOVA table** | Sum Sq | Mean Sq | dF | F-value | p-value |
| --- | --- | --- | --- | --- | --- |
| # leaves | 64.37 | 64.37 | 230.66 | 178.46 | <0.001 |
| length of longest leaf | 75.24 | 64.37 | 231.62 | 208.62 | <0.001 |

Note: Degrees of freedom (dF) estimated with Satterthwaite approximation
