# Supplementary material for: Plastic Population Effects and Conservative Leaf Traits in a Reciprocal Transplant Experiment Simulating Climate Warming in the Himalayas
Source: Front Plant Sci. 2018 Jul 30;9:1069. doi: 10.3389/fpls.2018.01069 (PMC6077237; doi:10.3389/fpls.2018.01069)
Supplement: Supplementary file 3 [file Table_3.DOCX]

**Supplementary Material S3**. Figures and likelihood ratio tests for vital rate regressions underlying the population models.

**Figures:**


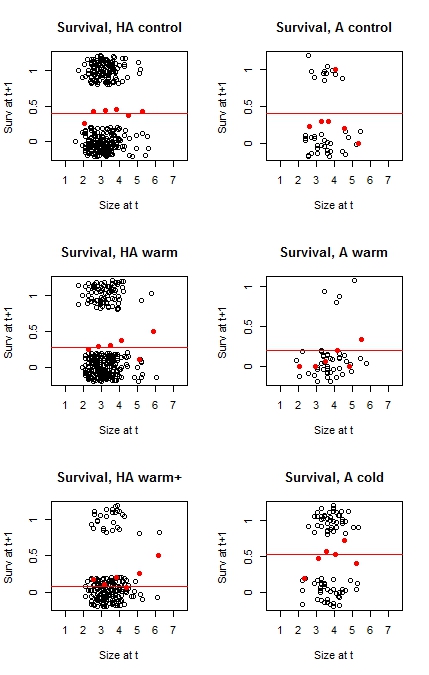


Figure S3.1. Models on survival probability (red lines) for climate transplants and controls in *Viola biflora*. Black open circles represent the original data. Closed red circles represent sequential means. Treatments ‘control’ and ‘warmer’ are given for both the high alpine (HA) and alpine (A) site.


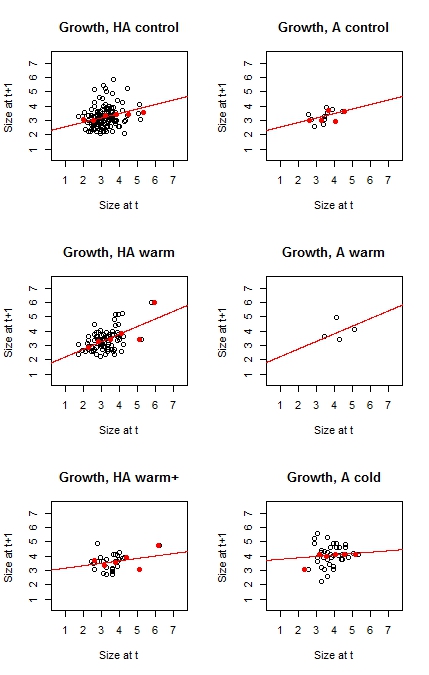


Figure S3.2. Models on growth of surviving individuals (red lines) for climate transplants and controls in *Viola biflora*. Black open circles represent the original data. Closed red circles represent sequential means. Treatments ‘control’ and ‘warmer’ are given for both the high alpine (HA) and alpine (A) site.


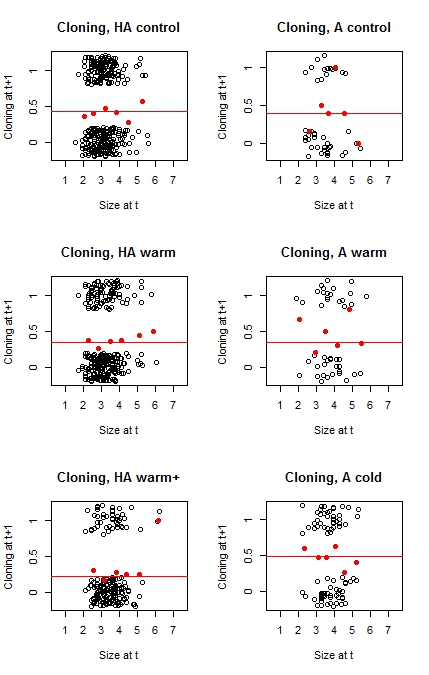


Figure S3.3. Models on whether or not an individual reproduced clonally (red lines) for climate transplants and controls in *Viola biflora*. Black open circles represent the original data. Closed red circles represent sequential means. Treatments ‘control’ and ‘warmer’ are given for both the high alpine (HA) and alpine (A) site.


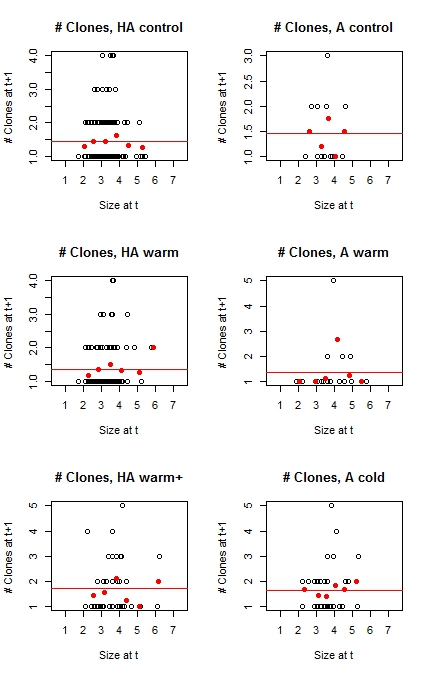


Figure S3.4. Models on number of clones produced by clonal individuals (red lines) for climate transplants and controls in *Viola biflora*. Black open circles represent the original data. Closed red circles represent sequential means. Treatments ‘control’ and ‘warmer’ are given for both the high alpine (HA) and alpine (A) site.


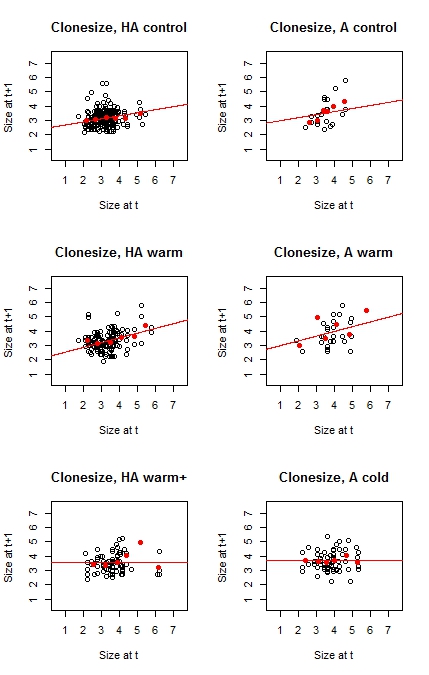


Figure S3.5. Models on relation between clone size and ‘mother’ size (red lines) for climate transplants and controls in *Viola biflora*. Black open circles represent the original data. Closed red circles represent sequential means. Treatments ‘control’ and ‘warmer’ are given for both the high alpine (HA) and alpine (A) site.


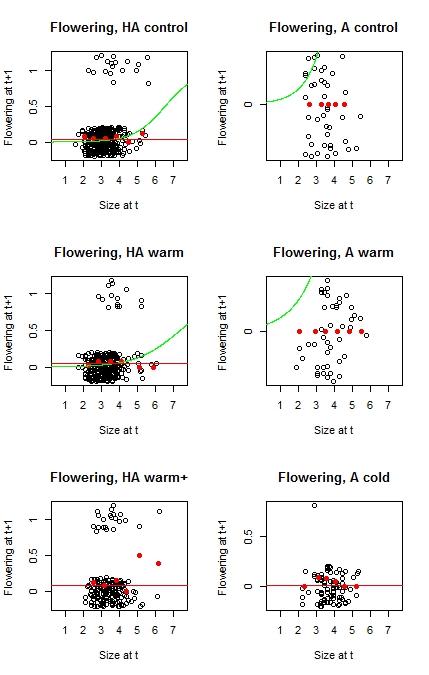


Figure S3.6. Models on flowering probability for climate transplants and controls in *Viola biflora*. Black open circles represent the original data. Closed red circles represent sequential means. Treatments ‘control’ and ‘warm’ are given for both the high alpine (HA) and alpine (A) site. Red lines represent the chosen models for building the fecundity matrices of the IPMs. Green lines represent the models identified as best fits by likelihood ration tests, but which were disregarded as they were poor fits for large plant sizes in the high alpine site and generally inappropriate for the alpine site.


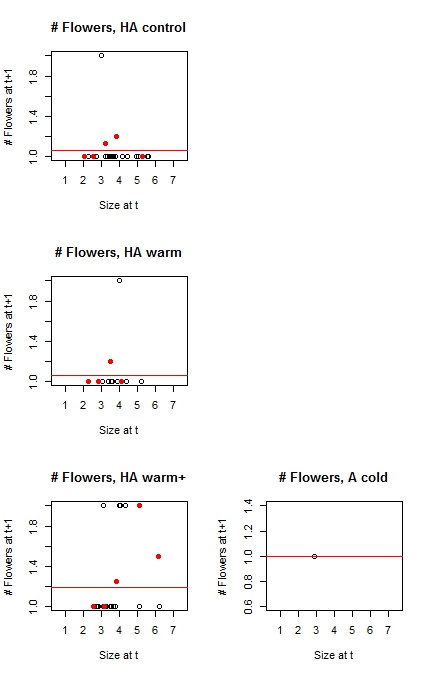


Figure S3.7. Models on number of flowers produced by flowering individuals (red lines) for climate transplants and controls in *Viola biflora*. Black open circles represent the original data. Closed red circles represent sequential means. There was only flower production in the high alpine site (HA), but none in the alpine site (see also Fig S3.6).

**Likelihood ration tests:**

#### growth ####

## control

model0: sizeNext ~ 1 + (1 | SiteTrans)

model1: sizeNext ~ size + (1 | SiteTrans)

model2: sizeNext ~ size + (size | SiteTrans)

Df AIC BIC logLik deviance Chisq Chi Df Pr(>Chisq)

model0 3 323.46 332.47 -158.73 317.46

model1 4 315.69 327.70 -153.84 307.69 9.7704 1 0.001773 **

model2 6 319.67 337.69 -153.84 307.67 0.0166 2 0.991740

## warmer

model0: sizeNext ~ 1 + (1 | SiteTrans)

model1: sizeNext ~ size + (1 | SiteTrans)

model2: sizeNext ~ size + (size | SiteTrans)

Df AIC BIC logLik deviance Chisq Chi Df Pr(>Chisq)

model0 3 194.68 202.05 -94.342 188.68

model1 4 171.11 180.93 -81.555 163.11 25.5736 1 4.258e-07 ***

model2 6 175.09 189.81 -81.544 163.09 0.0218 2 0.9891

## warmer+

model0: sizeNext ~ 1 + (1 | SiteTrans)

model1: sizeNext ~ size + (1 | SiteTrans)

model2: sizeNext ~ size + (size | SiteTrans)

Df AIC BIC logLik deviance Chisq Chi Df Pr(>Chisq)

model0 3 51.864 55.966 -22.932 45.864

model1 4 51.961 57.430 -21.980 43.961 1.9031 1 0.1677

model2 6 55.961 64.164 -21.980 43.961 0.0000 2 1.0000

## colder

model0: sizeNext ~ 1 + (1 | SiteTrans)

model1: sizeNext ~ size + (1 | SiteTrans)

model2: sizeNext ~ size + (size | SiteTrans)

Df AIC BIC logLik deviance Chisq Chi Df Pr(>Chisq)

model0 3 97.979 103.26 -45.989 91.979

model1 4 99.669 106.71 -45.834 91.669 0.3099 1 0.5777

model2 6 103.669 114.24 -45.834 91.669 0.0000 2 1.0000

#### survival ####

## control

model0: surv ~ 1 + (1 | SiteTrans)

model1: surv ~ size + (1 | SiteTrans)

model2: surv ~ size + (size | SiteTrans)

Df AIC BIC logLik deviance Chisq Chi Df Pr(>Chisq)

model0 2 504.88 512.72 -250.44 500.88

model1 3 506.78 518.54 -250.39 500.78 0.0976 1 0.7547

model2 5 510.78 530.38 -250.39 500.78 0.0000 2 1.0000

## warmer

model0: surv ~ 1 + (1 | SiteTrans)

model1: surv ~ size + (1 | SiteTrans)

model2: surv ~ size + (size | SiteTrans)

Df AIC BIC logLik deviance Chisq Chi Df Pr(>Chisq)

model0 2 379.37 386.95 -187.69 375.37

model1 3 381.37 392.73 -187.68 375.37 0.0031 1 0.9559

model2 5 385.35 404.28 -187.67 375.35 0.0217 2 0.9892

## warmer+

model0: surv ~ 1 + (1 | SiteTrans)

model1: surv ~ size + (1 | SiteTrans)

model2: surv ~ size + (size | SiteTrans)

Df AIC BIC logLik deviance Chisq Chi Df Pr(>Chisq)

model0 2 145.94 152.32 -70.968 141.94

model1 3 145.99 155.57 -69.993 139.99 1.9496 1 0.1626

model2 5 149.56 165.53 -69.780 139.56 0.4264 2 0.8080

## colder

Models:

model0: surv ~ 1 + (1 | SiteTrans)

model1: surv ~ size + (1 | SiteTrans)

model2: surv ~ size + (size | SiteTrans)

Df AIC BIC logLik deviance Chisq Chi Df Pr(>Chisq)

model0 2 117.48 122.29 -56.740 113.48

model1 3 118.16 125.38 -56.079 112.16 1.3227 1 0.2501

model2 5 122.10 134.13 -56.049 112.10 0.0606 2 0.9701

#### cloning ####

## control

Models:

model0: cloning ~ 1 + (1 | SiteTrans)

model1: cloning ~ size + (1 | SiteTrans)

model2: cloning ~ size + (size | SiteTrans)

Df AIC BIC logLik deviance Chisq Chi Df Pr(>Chisq)

model0 2 504.55 512.39 -250.28 500.55

model1 3 505.83 517.58 -249.91 499.83 0.7276 1 0.3937

model2 5 510.05 529.64 -250.02 500.05 0.0000 2 1.0000

## warmer

Models:

model0: cloning ~ 1 + (1 | SiteTrans)

model1: cloning ~ size + (1 | SiteTrans)

model2: cloning ~ size + (size | SiteTrans)

Df AIC BIC logLik deviance Chisq Chi Df Pr(>Chisq)

model0 2 424.76 432.33 -210.38 420.76

model1 3 425.09 436.45 -209.54 419.09 1.6703 1 0.1962

model2 5 429.09 448.02 -209.54 419.09 0.0000 2 1.0000

## warmer+

Models:

model0: cloning ~ 1 + (1 | SiteTrans)

model1: cloning ~ size + (1 | SiteTrans)

model2: cloning ~ size + (size | SiteTrans)

Df AIC BIC logLik deviance Chisq Chi Df Pr(>Chisq)

model0 2 181.46 187.84 -88.728 177.46

model1 3 180.81 190.39 -87.403 174.81 2.6494 1 0.1036

model2 5 183.43 199.40 -86.715 173.43 1.3761 2 0.5025

## colder

Models:

model0: cloning ~ 1 + (1 | SiteTrans)

model1: cloning ~ size + (1 | SiteTrans)

model2: cloning ~ size + (size | SiteTrans)

Df AIC BIC logLik deviance Chisq Chi Df Pr(>Chisq)

model0 2 117.63 122.44 -56.814 113.63

model1 3 119.02 126.24 -56.510 113.02 0.6083 1 0.4354

model2 5 123.02 135.05 -56.510 113.02 0.0000 2 1.0000

#### number of clones ####

## control

Analysis of Deviance Table

Model 1: clonesNext ~ size

Model 2: clonesNext ~ 1

Resid. Df Resid. Dev Df Deviance Pr(>Chi)

1 153 45.817

2 154 45.840 -1 -0.022292 0.8813

## warmer

Analysis of Deviance Table

Model 1: clonesNext ~ size

Model 2: clonesNext ~ 1

Resid. Df Resid. Dev Df Deviance Pr(>Chi)

1 111 33.268

2 112 34.112 -1 -0.84431 0.3582

## warmer+

Analysis of Deviance Table

Model 1: clonesNext ~ size

Model 2: clonesNext ~ 1

Resid. Df Resid. Dev Df Deviance Pr(>Chi)

1 43 22.245

2 44 22.831 -1 -0.58606 0.4439

## colder

Analysis of Deviance Table

Model 1: clonesNext ~ size

Model 2: clonesNext ~ 1

Resid. Df Resid. Dev Df Deviance Pr(>Chi)

1 38 17.976

2 39 18.161 -1 -0.1856 0.6666

#### clonesize ####

## control

model0: sizeNext ~ 1 + (1 | SiteTrans)

model1: sizeNext ~ size + (1 | SiteTrans)

model2: sizeNext ~ size + (size | SiteTrans)

Df AIC BIC logLik deviance Chisq Chi Df Pr(>Chisq)

model0 3 427.19 437.52 -210.60 421.19

model1 4 417.42 431.19 -204.71 409.42 11.7758 1 0.00060 ***

model2 6 414.33 434.99 -201.16 402.33 7.0864 2 0.02892 *

## warmer

model0: sizeNext ~ 1 + (1 | SiteTrans)

model1: sizeNext ~ size + (1 | SiteTrans)

model2: sizeNext ~ size + (size | SiteTrans)

Df AIC BIC logLik deviance Chisq Chi Df Pr(>Chisq)

model0 3 345.54 354.71 -169.77 339.54

model1 4 324.12 336.34 -158.06 316.12 23.420 1 1.302e-06 ***

model2 6 326.21 344.54 -157.10 314.21 1.912 2 0.3844

## warmer+

model0: sizeNext ~ 1 + (1 | SiteTrans)

model1: sizeNext ~ size + (1 | SiteTrans)

model2: sizeNext ~ size + (size | SiteTrans)

Df AIC BIC logLik deviance Chisq Chi Df Pr(>Chisq)

model0 3 163.68 170.78 -78.838 157.68

model1 4 164.87 174.34 -78.433 156.87 0.8093 1 0.3683

model2 6 168.87 183.08 -78.433 156.87 0.0000 2 1.0000

## colder

model0: sizeNext ~ 1 + (1 | SiteTrans)

model1: sizeNext ~ size + (1 | SiteTrans)

model2: sizeNext ~ size + (size | SiteTrans)

Df AIC BIC logLik deviance Chisq Chi Df Pr(>Chisq)

model0 3 142.93 149.63 -68.466 136.93

model1 4 143.99 152.93 -67.996 135.99 0.9403 1 0.3322

model2 6 147.99 161.39 -67.993 135.99 0.0064 2 0.9968

#### flowering ####

## control

model0: flowering ~ 1 + (1 | SiteTrans)

model1: flowering ~ size + (1 | SiteTrans)

model2: flowering ~ size + (size | SiteTrans)

Df AIC BIC logLik deviance Chisq Chi Df Pr(>Chisq)

model0 2 144.83 152.67 -70.416 140.83

model1 3 133.59 145.35 -63.796 127.59 13.2398 1 0.0002741 ***

model2 5 137.46 157.05 -63.728 127.46 0.1364 2 0.9340689

## warmer

model0: flowering ~ 1 + (1 | SiteTrans)

model1: flowering ~ size + (1 | SiteTrans)

model2: flowering ~ size + (size | SiteTrans)

Df AIC BIC logLik deviance Chisq Chi Df Pr(>Chisq)

model0 2 130.53 138.11 -63.266 126.53

model1 3 125.65 137.01 -59.827 119.65 6.8791 1 0.008721 **

model2 5 129.65 148.59 -59.826 119.65 0.0011 2 0.999440

## warmer+

model0: flowering ~ 1 + (1 | SiteTrans)

model1: flowering ~ size + (1 | SiteTrans)

model2: flowering ~ size + (size | SiteTrans)

Df AIC BIC logLik deviance Chisq Chi Df Pr(>Chisq)

model0 2 131.28 137.67 -63.643 127.28

model1 3 131.91 141.49 -62.957 125.91 1.3715 1 0.2416

model2 5 135.41 151.38 -62.708 125.42 0.4982 2 0.7795

## colder

model0: flowering ~ 1 + (1 | SiteTrans)

model1: flowering ~ size + (1 | SiteTrans)

model2: flowering ~ size + (size | SiteTrans)

Df AIC BIC logLik deviance Chisq Chi Df Pr(>Chisq)

model0 2 14.801 19.615 -5.4006 10.8012

model1 3 15.181 22.401 -4.5904 9.1809 1.6203 1 0.203

model2 5 19.181 31.215 -4.5904 9.1809 0.0000 2 1.000

#### number of flowers #####

## control

Analysis of Deviance Table

Model 1: flowers ~ size

Model 2: flowers ~ 1

Resid. Df Resid. Dev Df Deviance Pr(>Chi)

1 16 0.67891

2 17 0.71803 -1 -0.039124 0.8432

## warmer

Analysis of Deviance Table

Model 1: flowers ~ size

Model 2: flowers ~ 1

Resid. Df Resid. Dev Df Deviance Pr(>Chi)

1 14 0.70553

2 15 0.71135 -1 -0.0058206 0.9392

## warmer+

Analysis of Deviance Table

Model 1: flowers ~ size

Model 2: flowers ~ 1

Resid. Df Resid. Dev Df Deviance Pr(>Chi)

1 19 2.3022

2 20 2.3727 -1 -0.070441 0.7907

## colder

Analysis of Deviance Table

Model 1: flowers ~ size

Model 2: flowers ~ 1

Resid. Df Resid. Dev Df Deviance Pr(>Chi)

1 0 -2.1865e-21

2 0 -2.1865e-21 0 0
